# Factors Influencing Participation in Physiotherapy Services Following a Total Shoulder Replacement Surgery: A Cross‐Sectional Survey

**DOI:** 10.1002/hsr2.70830

**Published:** 2025-05-21

**Authors:** Rochelle Furtado, Marco Bandiera, Nevin Becker, Lisa Mitchell, Luca Paron, Tyler Shalansky, Dakota Turner‐Johnston, Joy C. MacDermid

**Affiliations:** ^1^ Department of Rehabilitation Sciences, Faculty of Health Science Western University London Ontario Canada; ^2^ School of Physical Therapy, Faculty of Health Science Western University London Ontario Canada; ^3^ Roth | McFarlane Hand and Upper Limb Centre St. Joseph's Hospital London Ontario Canada

**Keywords:** barriers, physiotherapy, rehabilitation, total shoulder replacement, upper extremity joint replacement

## Abstract

**Background:**

When designing appropriate rehabilitation programs after a shoulder replacement, it is critical to consider the multitude of factors that can influence a patient's participation. Therefore, this study aims to quantitively understand the factors that affect access and participation to physiotherapy services after a shoulder replacement in older adults.

**Methods:**

Our research team created an online of 58 questions, focusing on personal characteristics, geographic accessibility, socioeconomic status, preferences for care, social support, and cultural beliefs to understand potential barriers and facilitators to accessing services. Using a mixed‐methods approach, data was analyzed through quantitative descriptive statistics and interpretive descriptive methodology. Data was stratified by gender. Data collection took place in 2020–2022.

**Results:**

A total of 51 (53% women) people participated in this survey; with the average age of 64.6 (9.4) years old. Gender heavily influenced patients' preferences on accessing care and physiotherapy services. Social factors, economic factors and personal factors emerged as potential barriers to participation in physiotherapy for women. Patient expectations differed by gender, as women prioritize return to daily activities (93%), whereas men prioritized sport/recreational activities (85%). Finally, preferences for delivery of physiotherapy differed based on gender, as men prefer in person (77%) and women prefer virtual.

**Conclusion:**

This survey was able to investigate trends that influence participation to rehabilitation after a shoulder replacement, both quantitatively and qualitatively. This study is a starting point for future research to explore how factors such as gender roles and social expectations may affect individuals' participation in rehabilitation.

## 1 Introduction

As the need for joint replacement procedures increase within an aging population, so does the requirement to complete physiotherapy postoperatively [1]. Physiotherapy services have shown to be a critical factor in postoperative recovery after a shoulder replacement [1, 2]. However, current evidence reports that older adults face barriers when accessing and participating in physiotherapy services [3]. Not attending physiotherapy services after surgery results in slower recovery rates, decreased quality of life and ultimately not being able to gain the full benefits of the joint replacement procedure [2, 4].

While participation in physiotherapy services does rely on the willingness of the patient to attend, clinicians also play a key role in delivering equitable services, especially when rehabbing older adults [3, 5, 6]. Older adults are known to experience barriers such as additional co‐morbidities and less social support that can negatively attribute to lower exercise participation [3]. Previously reported qualitative studies exploring participation in physiotherapy after a hip or knee replacement, find that psychological barriers, sociodemographic barriers and a lack of social support can contribute to reduced participation in physiotherapy [7, 8]. More recent evidence also suggests that gender differences may influence adherence to exercise programs, with women being less likely to attend rehabilitation services compared to men due to receiving less social support to motivate their attendance [9]. However, since majority of the research being conducted is within participation to lower limb rehabilitation programs, there is a lack of research investigating participating in upper limb rehabilitation programs. Since recovery goals differ between upper limb and lower limb replacements, the current evidence may not be the same between both programs.

Therefore, when designing appropriate rehabilitation services after a shoulder replacement, it is critical to consider the multitude of factors that can influence a patient's participation within a rehabilitation program. From a recent qualitative study on adherence to upper extremity rehabilitation, participation barriers for patients included cost of treatment, patient‐provider relation (difference between therapist and patient understanding on what is important for treatment), and patients expecting the treating therapists to be an expert and fix their problem. The study concluded that assessing the identified factors can improve efficiency and effectiveness of clinical management and may enhance patient adherence to rehabilitation [10]. However, this study did not address whether gender also plays an influence towards participation. Having an increased awareness and understanding of these factors from a patient's viewpoint may improve participation in rehabilitation and further improve the care of shoulder replacement outcomes for all genders. Clinicians are continuingly seeking guidance on how to structure and deliver rehabilitation to facilitate shoulder replacement recovery, but may fail to achieve this if they do not address the patient's perspective of the current barriers that might exist in participation to rehabilitaion [11, 12]. Therefore to improve participation to shoulder replacement rehabilitation, the purpose of this study is to (1) explore the factors that affect access and participation to physiotherapy services after a shoulder replacement and (2) see if differences exist based on gender.

## 2 Methods

### 2.1 Study Design

Our study used a cross‐sectional survey of individuals residing in Ontario and have had a shoulder replacement, anatomical or reverse. This study was approved through the Western Research Ethics Board and Lawson Health Research Institute in London, Ontario.

### 2.2 Inclusion Criteria

Eligibility to complete the anonymized survey included: an individual who was over the age of 18, has previously had a shoulder replacement surgery, could speak and write in English, complete physiotherapy services post‐surgery and had access to an electronic device to participate in the survey.

### 2.3 Recruitment Process and Participation

Participants were recruited through both social media advertisements such as private Facebook groups, X and Instagram or in‐person though screening lists and posters in clinical waiting rooms. Student investigators approached patients in clinics and provided the recruitment posters for eligible participants. If participants had further questions about study participation, they were encouraged to contact the research assistant at the Hand and Upper Limb Centre Clinical Research Laboratory (HULC CRL) to answer any study related questions.

Study participation was voluntary and only required completion of the survey. No follow ups were required of participation after completion of the survey. No incentives were given for completing this study. Participants were required to read a Letter of Information, which detailed their study participation, how the data was stored, collected and analyzed, before completing the survey. The survey was completely anonymous and no personal information was collected to protect the identity of the participants. All data was collected and stored within an encrypted computer hard drive. Qualtrics does use Transport Layer Security (TLS) encryption for transmitted data to protect participants' responses during the submission process. Qualtrics is GDPR (*General Data Protection Regulation) compliant to meet privacy regulation standards*.

Access to any of the study related data, including participant's responses were restricted to authorized study personnel only. Overall results of the study were available upon request.

### 2.4 Survey

The survey used in this study was developed by a group of researchers and physiotherapists, based on guidance from literature, previously published work from our group [1, 12] and clinician experience with joint replacement produces. Creation of the survey took a few months as the group revised the versions according to structure and content. Once we created a version that was ready for review beyond the group, the survey was distributed for review among special interest group members including researchers, physical therapists and occupational therapists outside the research team (*n* = 10). We piloted a few surveys by our patient partner group comprising of 5 individuals receiving upper extremity care, including shoulder replacements. Feedback from these individuals helped to revise the final questionnaire. Production of the final survey took 4 months to complete.

The open survey consisted of 58 questions with the following sections: economic factors (8), geographical factors (6), health literacy factors (3), social factors (3), cultural factors (9), preferences of delivery (5), personal/identity factors (14), psychological factors (8) and patient expectations (2). At the end of the survey, demographics were collected. Anchors for the questions ranged from using a 5‐point Likert scale of strongly agree to strongly disagree. Some questions were also open text boxes for individuals to provide text answers. The only exception was the patient expectations section, which was measured on a 100‐point Visual Analogue scale. The survey was administered using commercially available software (Qualtrics, Seattle WA). Survey questions are presented within the Appendix (Table A1). Data was collected in August 2020 to August 2022 virtually. To account for minimal bias in our survey collection and reporting, we used the Checklist for Reporting Results of Internet E‐Surveys (CHERRIES); checklist results are presented within the Appendix (Table A1) [13].

### 2.5 Data Analysis

Descriptive statistics were reported for each individual survey question, including means and standard deviations. Using Sex and Gender Equity in Research (SAGER) guidelines [14], data was disaggregated by sex and gender when possible. Since only 10% of data was missing from the survey, we decided to remove missing data from the analysis. All data analyses were stored and performed on SPSS software.

Qualitative data was also extracted from the open text box answers within the survey. Meaningful themes were co‐created from the data using a thematic analysis approach as detailed by Thorne [15]. This involves repetitive reading of the transcripts and coding of emerging themes from the data. The intention of interpretive description is to develop clinically useful information. Therefore, the data was organized into themes relevant to the data. This process was inductive, and data was coded through the open coding method. Using the constant comparative method, codes and processes were constantly compared within each participant's data and across participants to form categories and themes. Themes were finalized during meetings between all authors.

## 3 Results

A total of 60 participants initiated the survey but 51 patients fully completed the survey. Participants' demographic information is shown in Table 1. A summary of the results stratified by gender is presented in Table 2.

**Table 1:** Demographic information about survey participants (*n* = 51).

	Patients
Age [mean (standard deviation)]	64.6 (9.4)
Gender	
Woman	53%
Man	47%
Type of shoulder replacement	
Anatomical replacement	23%
Reverse shoulder replacement	77%
Caregiver after surgery?	
Yes	82%
No	18%
Highest level of education	
Less than high school degree	4%
High school graduate	4%
Some college/university but no degree	8%
College	8%
University—Bachelor's	13%
University—Masters	15%
Doctoral degree	0%
Professional degree (JD, MD)	3%
Martial status	
Single	2%
Married	47%
Separated	4%
Divorced	6%
Widowed	0%

**Table 2:** Summary of results stratified by gender.

Factor	Men	Women
Economic	Men are more willing to pay out of pocket for physiotherapy	Women were not willing to pay out of pocket for physiotherapy or extra equipment
Geographical/environmental	No differences between genders	No differences between genders
Health literacy	No differences between genders	No differences between genders
Social support	Men expressed they have a strong support system to attend physiotherapy	Women report having less social support to attend physiotherapy and more embarrassed to let others know they are attending physiotherapy
Cultural	No differences between genders	No differences between genders
Personal	Men expressed exercising was already a part of their routine and easier to integrate physiotherapy into	Women expressed exercising was not already apart of their routine and harder to integrate physiotherapy into
Identity	Men did not express a concern for the look of their therapist	Women preferred a younger therapist
Psychological	Men preferred exercising alone	Women preferred group exercise
Patient expectations	The most important expectation from participating in physiotherapy was improved function, independence, return to work and lower chance of re‐injury for men	The most important expectation for women was the ability to perform activities of daily living, and the lowest ranked expectation was return to work

### 3.1 Economic Factors

Generally, access to physiotherapy was not an issue for majority of participants as over 76.8% of people agreed they were easily able to access physiotherapy services after surgery. However, financial resources for physiotherapy coverage did vary amongst participations with 11.4% relying on the Ontario Health Insurance Plan, 34.3% having personal coverage/insurance, 11.4% needing to pay out of pocket and 34.3% reporting other means of coverage. When asked if participants would be willing to pay for physiotherapy services if needed to, majority (69.4%) were able to, while 16.7% participants indicated they might be able to and 13.9% reported they would not pay. Those who reported they would not be able to pay were all women. When asked what participants were willing to spend on one treatment session most participants (56.5%) said $50 or under, 28% said $80 or under, 12% reported they are willing to spend $100 or under and only 4% said they would pay over $100. Lastly, participants were asked if they could afford additional exercise equipment to continue with home exercises on their own. Many of the participants (63.6%) indicated they were able to afford additional equipment at home, 24.2% did not agree or disagree and 11% indicated they could not afford additional equipment at home. When stratified by gender, of those that indicated they could not afford extra equipment, only one of them was a man and the rest were women.

When analyzing the qualitative data within this section, two prominent themes were co‐constructed from the data. The first theme was *Experience versus Costs*, where some participants expressed wanting an experienced physiotherapist that specialized in shoulder replacement rehabilitation, but that may result in higher treatment costs and/or limited access to these specialists. The second theme was *Not Making Ends Meet*, where participants expressed that even with insurance coverage, the amount of coverage was limited and may not cover the entirety of the treatment, for example, insurance only covering 50% of the appointment, or having a set budget for physiotherapy sessions in a year.

### 3.2 Geographical/Environmental Factors

Most participants (73.3%) lived in smaller cities/towns (< 500,000 people). Some indicated (26.8%) they lived in urban cities (> 500,000 people) and 16.7% of participants lived in rural areas (< 5000 people). Distance to a clinic was not an issue to majority of people (80.6%), as they reported they lived 10 km of a physiotherapy clinic, and 19.3% lived greater than 10 km away from a clinic. When asked about transportation methods, most (91%) reported they had access to a personal vehicle such as a car to be able to attend physiotherapy, while 6% indicated they used public transportation and 3% indicated they walked to physiotherapy. When asked whether environmental conditions, such as heavy rain or snowfall impacted their ability to go attend physiotherapy, opinions were split between 38% agreeing it impacted them going to physiotherapy, 38% not agreeing it had an impact and 24% neither agreeing nor disagreeing.

### 3.3 Health Literacy Factors

Health literacy was not a major barrier towards participating in physiotherapy services. Most participants (86.7%) felt confident with the information on physiotherapy provided to them from their surgeon. Participants also felt they could confidently read information about their health (90%) and apply that information (90%) towards their own health and well‐being.

### 3.4 Social Factors

Overall, social factors were heavily influenced by gender differences. While many participants indicated a strong social support from friends, family and their healthcare team to attend physiotherapy, there was a noticeable between gender, when reporting on if they did have social support to attend physiotherapy. From the 10% that reported they did not have strong support to attend physiotherapy, their gender was women. Conversely, all men within our survey reported having strong support from friends/family to participate in rehabilitation. When identifying if a participant was embarrassed to tell their family members or friends, they attend physiotherapy, only women (8%) indicated they were embarrassed to tell their family members or friends.

### 3.5 Preferences of Physiotherapy Delivery

Preferences varied amongst how individuals preferred physiotherapy to be delivered to them. Firstly, many participants prefer attending small private physiotherapy clinics (48%), while 16% prefer a hospital setting for care and 35% do not have a preference. Interestingly, 77% of participants wanted face‐to‐face appointments with their physiotherapist rather than virtual. Only women indicated they preferred a virtual option or did not have a preference to virtual/in person, while all men said they prefer in person physiotherapy. Delivery options for exercise prescription also varied in the population where 24.1% reported that they preferred a paper handout, 34.5% preferred a website, 37.9% preferred a video of the exercise and 3.4% preferred a CD/DVD method. Most college and university educated participants preferred videos and website platforms over paper handouts.

### 3.6 Cultural Factors

English was the primary spoken language amongst participants in this survey, and most felt they could communicate their concerns (87%) and/or understand all instructions (97%) from their physiotherapist. When asked if their physiotherapist considers their culture, beliefs and goals when creating their physiotherapy treatment plans, slightly more than half agreed (57%) reported yes, 36% reported neither agree nor disagree and 7% disagreed. All the individuals that reported that they do not feel their therapist considers their culture, beliefs or goals to create a treatment plan were women. When asked if participants felt more comfortable with a physiotherapist that does share similar beliefs as them, 69% neither agreed nor disagreed with this statement while only 27% said they agreed.

When analyzing the qualitative data within this section, one theme emerged from the data. The theme *Communication is key*, described that some participants felt more comfort because they spoke the same language as their therapist, and so their concerns about treatment or recovery were easy to communicate.

### 3.7 Personal Factors

When understanding what motivates people to attend physiotherapy, 59% indicated that since their surgery was on their dominant hand, they are more likely to attend physiotherapy. Many participants who were already exercising (55%) felt it was easier to integrate physiotherapy into their routine. However, 20% of individuals, all who were women, expressed that exercise was not already part of their routine. Additionally, while 82% expressed not having too many personal commitments to limit their participation in physiotherapy, 3% (all women) indicated their personal commitments limited their participation in physiotherapy. When participants were furthered prompted on how much time they can spend for physiotherapy, answers ranged from 30 min a day to 2 h a day.

When analyzing the qualitative data within this section, one theme was co‐constructed from the data. The theme *Caregiver first*, described that a few participants felt their caregiving duties outweighed their ability to attend physiotherapy services. For example, one participant said, *“I have 2 jobs, 5 children and 1 spouse that take up my personal time.”*

### 3.8 Identity Factors

Our survey asked participants their opinions of their physiotherapist's appearance, and if this influenced their participation to physiotherapy. Our results indicated that majority of individuals were neutral (62%) to the opinion that their physiotherapy should be a young/health individual. Of the 20% that indicated they would prefer their physiotherapist to be a young and healthy individual, 17% were women and 3% were men. Our results also indicated that majority of individuals were either neutral (69%) or disagreed (28%) to the opinion that their physiotherapist should be the same sex/gender as them.

Our survey also asked participants their opinions of the physiotherapy clinic they attended. Results indicated that many participants (75%) enjoyed the environment of their clinic, while 7% did not enjoy their clinic environment. Many also indicated that they were either neutral to (72%) or did not need (24%) the other patients that attended their clinic to have a similar appearance as them.

When analyzing the qualitative data within this section, one theme emerged from the data. The theme *Knowledge is Power*, described majority of participants wanted their physiotherapist to be experienced and knowledgeable about their condition and treatment, and appearance of the therapist was not something important to them. For example, one participant said, *“It is about their education level, not their appearance.”*

### 3.9 Psychological Factors

Generally, most participants (86%) agreed that physiotherapy did help them recovery after surgery. There was an even split as to people who prefer to exercise alone (59%) versus people who like group exercise (58%). Women indicated they would be more willing to participate in group exercise than men. However, 21% of participants did indicate they feel anxious or stressed to complete their exercise program at home by themselves. While 24% of individuals reported pain levels from stopping their participation in their home exercise programs.

### 3.10 Patient Expectations

Participants' expectations regarding physiotherapy treatment after surgery was captured on a scale of 0–100. Mean scores of participants' responses by gender are described in Table 3. The most important expectation from participating in physiotherapy was improved function, independence, return to work and lower chance of re‐injury for men. The most important expectation for women was the ability to perform activities of daily living, and the lowest ranked expectation was return to work.

**Table 3:** Participants' reported scores of their expectations forparticipating in physiotherapy (*n* = 51).

Type of expectations	Women [mean (SD)]	Men [mean (SD)]
Relief of pain	85.2 (20.1)	85 (21.1)
Improved function	91.2 (11.8)	100 (0)
Improved ability to perform activities of daily living	93.1 (10.8)	87.3 (26.8)
Improved independence	91.1 (17.2)	100 (0)
Improved participation in recreational activities	72.9 (27.9)	85 (21.2)
Return to work	67.7 (41.2)	100 (0)
Lower chance of re‐injury	85.4 (21.8)	100 (0)

*Note:* Scores are reported by means and standard deviations (SD).

## 4 Discussion

Our study identified that participation in physiotherapy services after a shoulder replacement may be influenced by gender roles and expectations. Despite our study having a smaller sample size, social factors, economic factors, and personal factors emerged as potential barriers to participation in physiotherapy for women. Patient expectations also differed when analyzed by gender, as women prioritize return to daily activities rather than sport/recreational activities as reported by men. Finally, preferences for delivery of physiotherapy differed based on gender, as men prefer in person and women prefer virtual. Financial barriers and caregiving roles may be larger barriers for women than men. While this study is a starting point, future studies should qualitatively investigate gender differences to participation in rehabilitation after a joint replacement, to better understand how gender influences participation to rehabilitation.

One of the largest barriers reported in our population to physiotherapy participation was social factors. Our study investigated social factors such as social networks and their influences on older adults participating in physiotherapy. While many participants indicated they did have strong social networks, those that did not, were women. In studies on cardiac rehabilitation attendance, having good social networks was shown to lead to better rehabilitation attendance [16, 17, 18]. However, women within cardiac rehabilitation more commonly report struggling with depression, anxiety, stress and less familial support. This negatively leads to women having lower adherence to self‐care and attendance to medical appointments [19]. While this phenomenon has not been commonly studied in postoperative joint replacement rehabilitation, a solution reported by cardiac rehabilitation is to have an option of group therapy [16]. Group therapy promotes a sense of community and support that may empower women to continue to participate in exercise. In 2009, Coulter and colleagues investigated group‐based exercises versus home based on a population of community dwellers that had a hip or knee replacement [20]. Quantitative findings indicated no differences in recovery between the two groups and so, further investigation of this method is warranted for post shoulder replacement surgery and if it will improve women' participation in rehabilitation [20].

Economical factors continue to limit participation in physiotherapy especially in older adults. As demonstrated within our study, women may have had less finances to participate in physiotherapy if they had to pay out of pocket or required additional rehabilitation equipment. As demonstrated in cardiac rehabilitation, women tend to have lower economic means than men, which can affect their participation [16]. One solution to help alleviate the gender disparities of finances, is the model of bundled care. As shown in literature, bundled care is a funding model developed to promote integration in healthcare delivery, drive high‐quality care, and improve patient outcomes [21]. As shown in the study by Chen and colleagues [21], participants who experienced this pathway found success in transitioning from acute care services to out‐patient physiotherapy, while not having to worry about financial costs. While bundled care is still being implemented mainly for hip and knee replacements due to their extensive volume of procedures yearly, a bundled model of care for shoulder replacements has begun in some sites [22]. Future studies should investigate gendered differences to participation in bundled care services, once more established across Canada.

Personal factors were another barrier that may have been influenced by gender. Women participants indicated that they had less time to commit to exercise and physiotherapy compared to men. As shown in a previous work by our group, older women who were in a relationship and experienced additional household members (i.e., dependent children), were dealing with more changes in unpaid work roles during the pandemic, when compared to other groups of individuals [23]. Our data in the present study was collected during the peak years of the pandemic and may explain why women faced even more caregiving duties that could have limited their participation in exercises and rehabilitation. A strategy to increase participation may be to have more virtual options of physiotherapy [24]. This might provide a way for women who cannot leave the house since they are tending to a child or sick family member, but still ensures they can get access to healthcare for themselves [25]. This choice was also evident in our survey, as many women indicated they would prefer a virtual option to therapy. However, this option may be less useful for men.

Lastly, although patients expressed high expectations for shoulder replacement surgery, patient expectations of physiotherapy were ranked differently based on gender. Our results indicated that men prioritized function and return to sport more highly, compared to women who prioritize return to daily activities. As previously mentioned, this may be attributed to role differences between older men and women, particularly during the pandemic [23]. However, trends in patient expectations from our study, were aligned with previous studies of patient expectations of receiving a shoulder replacement. As shown previously, in a prospective study of 63 patients undergoing a shoulder replacement by Jawa et al. [26], men patients had identified their top preoperative expectations as participation in sports, sleeping through the night, and maintaining employment. Conversely, patients that were women had identified preoperative expectations of independently performing household chores and their daily routine, which differed significantly from men (*p* < 0.01). This suggests that gendered role expectations around home and family responsibilities were the main reason for gendered differences, and that these were not specific to changes that may have occurred during the pandemic [27].

Our study highlights that gender may be a factor towards participation in shoulder rehabilitation [27, 28, 29]. While limited research is present on whether gender influences participation in musculoskeletal rehabilitation, women are more heavily impacted by arthritis that prompt the need for shoulder replacements. Understanding they may have more financial and role‐related barriers to attending physiotherapy should be considered. However, designing programs that meet the different needs of both genders is needed to improve the patient experience, outcomes and equity. Therefore, it is critical that physiotherapists and researchers re‐envision how to provide gender transformative rehabilitation services to optimize recovery following total shoulder arthroplasty.

### 4.1 Limitations

Despite having a moderate sample size, our survey was able to investigate trends that influence participation to rehabilitation after a shoulder replacement, both quantitatively and qualitatively. With having more women than men respond, we were able to identify some emerging gender differences that impact participation. Our study is a starting point for future research to explore how factors such as gender roles and social expectations may affect individuals' participation in rehabilitation. However, it is important to note that the survey participants were only located in Ontario, Canada, therefore, responses may change dependent on province, as access to healthcare varies by province in Canada, and across different countries. Future studies should expand data collection across the country to see if the results are equally across the country. Our sample size was majority persons of Caucasian descent, living in more urban settings. This may attribute as to why so many participants indicated they had a car and/or lived near a physiotherapy clinic. To help engage with more people of different ethnicities and lifestyles, we recruited from social media such as Facebook groups etc. to capture voices within different communities. However, further work should collect different ethnicities to see if similar trends emerge as within our study or if ethnicity plays a role in participation to rehabilitation. Finally, we did not capture work status in our survey. However from literature, majority of people who undergo a shoulder replacement are above the age of 65 indicating they are most likely retired. We hypothesize that is why some people indicated they can spend more than 30 min towards exercises in a day, however future research should collect information on work status to see if job occupation type and status influences participation.

## 5 Conclusion

Patient preferences and the factors that affect their participation to rehabilitation may be influenced by gender. While this study is a starting point, future work needs to investigate how to provide gender transformative rehabilitation services to optimize patient experience, outcomes and equity following total shoulder replacements.

## Author Contributions

**Rochelle Furtado:** conceptualization, data curation, formal analysis, methodology, project administration, resources, writing – original draft, writing – review and editing. **Marco Bandiera:** data curation, writing – original draft. **Nevin Becker:** data curation, writing – original draft, writing – review and editing. **Lisa Mitchell:** methodology, writing – original draft. **Luca Paron:** methodology, writing – original draft. **Tyler Shalansky:** methodology, writing – original draft. **Dakota Turner‐Johnston:** data curation, writing – original draft, writing – review and editing. **Joy C. MacDermid:** supervision, validation, writing – original draft, writing – review and editing.

## Ethics Statement

This study was approved by the Western Research Ethics Board and Lawson Research.

## Conflicts of Interest

The authors declare no conflicts of interest.

## Transparency Statement

The lead author Rochelle Furtado affirms that this manuscript is an honest, accurate, and transparent account of the study being reported; that no important aspects of the study have been omitted; and that any discrepancies from the study as planned (and, if relevant, registered) have been explained.

## Data Availability

The data that support the findings of this study are available from the corresponding author upon reasonable request.

## Appendix A

### A.1

**Table A1:** Checklist for Reporting Results of Internet E‐Surveys (CHERRIES).

Checklist item	Explanation	Page number
Describe survey design	Describe target population, sample frame. Is the sample a convenience sample? (In “open” surveys this is most likely)	5
IRB approval	Mention whether the study has been approved by an IRB	4
Informed consent	Describe the informed consent process. Where were the participants told the length of time of the survey, which data were stored and where and for how long, who the investigator was, and the purpose of the study?	4
Data protection	If any personal information was collected or stored, describe what mechanisms were used to protect unauthorized access	5
Development and testing	State how the survey was developed, including whether the usability and technical functionality of the electronic questionnaire had been tested before fielding the questionnaire	5
Open survey versus closed survey	An “open survey” is a survey open for each visitor of a site, while a closed survey is only open to a sample which the investigator knows (password‐protected survey)	5
Contact mode	Indicate whether or not the initial contact with the potential participants was made on the Internet. (Investigators may also send out questionnaires by mail and allow for Web‐based data entry)	4
Advertising the survey	How/where was the survey announced or advertised? Some examples are offline media (newspapers), or online (mailing lists – If yes, which ones?) or banner ads (Where were these banner ads posted and what did they look like?). It is important to know the wording of the announcement as it will heavily influence who chooses to participate. Ideally the survey announcement should be published as an appendix	4
Web/E‐mail	State the type of e‐survey (e.g., one posted on a Website, or one sent out through e‐mail). If it is an e‐mail survey, were the responses entered manually into a database, or was there an automatic method for capturing responses?	4
Context	Describe the Website (for mailing list/newsgroup) in which the survey was posted. What is the Website about, who is visiting it, what are visitors normally looking for? Discuss to what degree the content of the Website could pre‐select the sample or influence the results. For example, a survey about vaccination on an anti‐immunization Website will have different results from a Web survey conducted on a government Website	5
Mandatory/voluntary	Was it a mandatory survey to be filled in by every visitor who wanted to enter the Website, or was it a voluntary survey?	4
Incentives	Were any incentives offered (e.g., monetary, prizes, or non‐monetary incentives such as an offer to provide the survey results)?	5
Time/Date	In what timeframe were the data collected?	5
Randomization of items or questionnaires	To prevent biases items can be randomized or alternated	N/A
Adaptive questioning	Use adaptive questioning (certain items, or only conditionally displayed based on responses to other items) to reduce number and complexity of the questions	5
Number of items	What was the number of questionnaire items per page? The number of items is an important factor for the completion rate	5
Number of screens (pages)	Over how many pages was the questionnaire distributed? The number of items is an important factor for the completion rate	N/A
Completeness check	It is technically possible to do consistency or completeness checks before the questionnaire is submitted. Was this done, and if “yes”, how (usually JAVAScript)? An alternative is to check for completeness after the questionnaire has been submitted (and highlight mandatory items). If this has been done, it should be reported. All items should provide a non‐response option such as “not applicable” or “rather not say”, and selection of one response option should be enforced	N/A
Review step	State whether respondents were able to review and change their answers (e.g., through a Back button or a Review step which displays a summary of the responses and asks the respondents if they are correct)	5
Unique site visitor	If you provide view rates or participation rates, you need to define how you determined a unique visitor. There are different techniques available, based on IP addresses or cookies or both	n/a
View rate (Ratio of unique survey visitors/unique site visitors)	Requires counting unique visitors to the first page of the survey, divided by the number of unique site visitors (not page views!). It is not unusual to have view rates of less than 0.1% if the survey is voluntary	n/a
Participation rate (Ratio of unique visitors who agreed to participate/unique first survey page visitors)	Count the unique number of people who filled in the first survey page (or agreed to participate, for example by checking a checkbox), divided by visitors who visit the first page of the survey (or the informed consents page, if present). This can also be called “recruitment” rate	7
Completion rate (Ratio of users who finished the survey/users who agreed to participate)	The number of people submitting the last questionnaire page, divided by the number of people who agreed to participate (or submitted the first survey page). This is only relevant if there is a separate “informed consent” page or if the survey goes over several pages. This is a measure for attrition. Note that “completion” can involve leaving questionnaire items blank. This is not a measure for how completely questionnaires were filled in. (If you need a measure for this, use the word “completeness rate”)	7
Cookies used	Indicate whether cookies were used to assign a unique user identifier to each client computer. If so, mention the page on which the cookie was set and read, and how long the cookie was valid. Were duplicate entries avoided by preventing users access to the survey twice; or were duplicate database entries having the same user ID eliminated before analysis? In the latter case, which entries were kept for analysis (e.g., the first entry or the most recent)?	Not allowed due to ethics recommendations
IP check	Indicate whether the IP address of the client computer was used to identify potential duplicate entries from the same user. If so, mention the period of time for which no two entries from the same IP address were allowed (e.g., 24 h). Were duplicate entries avoided by preventing users with the same IP address access to the survey twice; or were duplicate database entries having the same IP address within a given period of time eliminated before analysis? If the latter, which entries were kept for analysis (e.g., the first entry or the most recent)?	Not allowed due to ethics recommendations
Log file analysis	Indicate whether other techniques to analyze the log file for identification of multiple entries were used. If so, please describe	N/A
Registration	In “closed” (non‐open) surveys, users need to login first and it is easier to prevent duplicate entries from the same user. Describe how this was done. For example, was the survey never displayed a second time once the user had filled it in, or was the username stored together with the survey results and later eliminated? If the latter, which entries were kept for analysis (e.g., the first entry or the most recent)?	N/A
Handling of incomplete questionnaires	Were only completed questionnaires analyzed? Were questionnaires which terminated early (where, for example, users did not go through all questionnaire pages) also analyzed?	6
Questionnaires submitted with an atypical timestamp	Some investigators may measure the time people needed to fill in a questionnaire and exclude questionnaires that were submitted too soon. Specify the timeframe that was used as a cut‐off point and describe how this point was determined	N/A
Statistical correction	Indicate whether any methods such as weighting of items or propensity scores have been used to adjust for the non‐representative sample; if so, please describe the methods	6
